# Criminal Trajectories Across the Dementia Timeline—A Nationwide Finnish Register Study

**DOI:** 10.1002/gps.70131

**Published:** 2025-07-16

**Authors:** Milena Ginters, Tiina Talaslahti, Hannu Kautiainen, Risto Vataja, Anniina Palm, Henrik Elonheimo, Jaana Suvisaari, Nina Lindberg, Hannu Koponen

**Affiliations:** ^1^ Psychiatry University of Helsinki and Helsinki University Hospital Helsinki Finland; ^2^ Primary Health Care Unit Kuopio University Hospital Kuopio Finland and Folkhälsan Research Center Helsinki Finland; ^3^ University of Helsinki Helsinki Finland; ^4^ Criminology and Restorative Justice University of Turku Turku Finland; ^5^ Finnish Institute for Health and Welfare Mental Health Team Helsinki Finland

**Keywords:** criminal behaviour, criminality, dementia, neurocognitive disorders, neuropsychiatric symptoms, older population

## Abstract

**Objectives:**

The aim of this longitudinal register study was to examine the crime counts, crime incidence and timing of criminal activity in relation to the diagnosis of Alzheimer's disease (AD), frontotemporal dementia (FTD) and Lewy body dementias (LBD). The objective was to analyse the associated risk factors and risk relations among the offender categories.

**Methods:**

We collected register data from Finnish nationwide registers (Finnish Care Register for Health Care and Finnish National Police Register) between 1998 and 2015. Mortality statistics were collected until the end of 2018 (Statistics Finland). Our study included a total of 92,189 patients, of whom 80,540 had AD, 1059 had FTD, and 10,590 had LBD. We examined the crimes committed by the study population before and after diagnosis. In the follow‐up, we primarily focussed on the 4‐year period and secondarily on the 10‐year period after diagnosis. First, we measured the post‐diagnostic crime rates in patients who had committed crimes before diagnosis and in those who had not; we also calculated the incidence rate ratio (IRR) of post‐diagnosis crimes in these groups. Second, we calculated the cumulative incidence of first post‐diagnoses crimes and investigated the risk of the first post‐diagnoses crime by calculating the adjusted subhazard ratio (sHR) in pre‐diagnosis offenders and non‐offenders.

**Results:**

Most of the study population did not exhibit criminal behaviour before or after diagnosis. However, individuals who had engaged in criminal behaviour before diagnosis also showed a higher incidence of criminal activity in the 4‐year period after diagnosis compared to patients with no criminal history prior to diagnosis. They also showed a steep increase in the cumulative incidence of the first post‐diagnosis crime, particularly in the first 2 years after diagnosis. The age‐ and sex‐adjusted sHR for the occurrence of the first post‐diagnosis crime was 4.42 (95% confidence interval: 3.83–5.11) in AD, 4.36 (2.15–8.83) in FTD and 4.87 (3.88–6.12) in LBD in pre‐diagnosis offenders versus non‐offenders.

**Conclusions:**

Individuals with a history of criminal behaviour before diagnosis of a neurocognitive disorder showed higher rates and a higher risk of future criminal activity. The cumulative incidence rose steeply during the first 2 years after diagnosis and after more gradually. Criminal activity closer to the time of diagnosis, especially 1 year prior, was the strongest predictor in increasing the risk of future criminal behaviour.

## Key Points

- Although criminal incidents were infrequent overall, patients who had committed crimes before their diagnosis exhibited a higher rate of criminal activity after diagnosis.
- Among patients with a history of pre‐diagnosis criminal behaviour, the incidence of subsequent criminal activity was approximately four times higher in those with FTD and nearly six times higher in those with AD and LBD, compared to patients without a criminal history.
- The cumulative incidence of the first crime post‐diagnosis increased steadily over the 10‐year follow‐up period.
- Criminal activity occurring closer to the time before diagnosis, was the most significant predictor of future criminal behaviour and should be taken into account in the treatment of this population.

## 1 Introduction

Neurocognitive disorders are common in the ageing population [1] and become increasingly prevalent with age [2]. As the global population of older individuals is projected to rise in the coming decades, the total number of individuals with dementia is expected to reach around 152 million in 2050 [3, 4]. Neurodegenerative disorders are associated with cognitive changes and neuropsychiatric symptoms and pose a risk for socially inappropriate or even delinquent behaviour. Novel criminal behaviour emerging at a later age may thus indicate the onset of an underlying neurocognitive disorder [5, 6]. Despite the high prevalence of neurocognitive disorders, there is limited research on dementia‐related criminal behaviour, particularly regarding the incidence of criminal behaviour in relation to the timing of diagnosis and disease progression [6, 7, 8].

As part of our Criminal Behaviour in Neurocognitive Disorders (CRICO) study [5, 9], we have previously explored criminal behaviour separately before and after diagnosis of a neurocognitive disorder [5, 9]. In this study, by using a longitudinal register study design, our aim was to explore the incidence of criminal activity with a focus on its timing in relation to a diagnosis of neurocognitive disorder of Alzheimer's disease (AD), frontotemporal dementia (FTD), and Lewy body dementias (LBD, including dementia with Lewy bodies and Parkinson's disease dementia). We aimed to determine whether individuals who committed crimes before the neurocognitive diagnosis continued their criminal activity after diagnosis. Additionally, we analysed risk associations related to the continuation of the post‐diagnosis criminal behaviour across different diagnostic categories.

## 2 Material and Methods

### 2.1 Ethical Considerations and Approval

The study was conducted as a register study; thus, the participants are not identifiable and not contacted, nor was any informed consent required. The study protocol was approved by the Coordinating Ethics Committee of Helsinki University Hospital (no 186/13/03/00/16).

## 3 Setting

### 3.1 Study Design and Population

The study included all Finnish individuals who were diagnosed with Alzheimer's disease (AD), frontotemporal dementia (FTD), or Lewy body dementias (LBD; including dementia with Lewy bodies and Parkinson's disease dementia) and were aged 40 or more at diagnosis. We chose to exclude people under 40 years, because the prevalence of early‐onset dementias is low in that population [10, 11]. The data of the study population were collected from the Finnish nationwide registers and included a total of 92,189 patients, of whom 80,540 had AD, 1059 FTD, and 10,590 LBD (Table 1). These individuals were monitored during the follow‐up period for maximum of 10 years after diagnosis, until their death or until the end of data collection period. Both sexes were combined, because otherwise the subgroup of women would have been too small.

**TABLE 1:** Characteristics of patients with and without criminal activity in the four groups 4 years before and 4 years after diagnosis of a neurocognitive disorder.

4 years before diagnosis	No crimes before diagnosis (−)		Crimes before diagnosis (+)		*p*‐value	
4 years after diagnosis	No crimes at all (non‐offenders) (−/−)	Crimes only after diagnosis (post‐diagnosis offenders) (−/+)	Crimes only before diagnosis (discontinuers) (+/−)	Crimes before and after diagnosis (continuers) (+/+)	Crude	Adjusted^a^
AD						
Number of persons	75,556	618	4016	350		
Women, *n* (%)	52,034 (69)	159 (26)	797 (20)	38 (11)	*p* < 0.001	
Mean age at diagnosis, mean (SD)	81 (7)	74 (8)	77 (7)	72 (9)	*p* < 0.001	
Person‐years followed after diagnosis	249,281	3092	11,888	1544		
Number of deaths	43,523	220	1830	84		
10‐year survival, % (95% CI)^b^	13.6 (13.2–14.0)	45.2 (38.9–51.3)	12.9 (10.7–15.2)	49.7 (39.6–59.3)	< 0.001	< 0.001
FTD						
Number of persons	899	22	116	22		
Women, *n* (%)	564 (63)	8 (36)	28 (24)	3 (14)	< 0.001	
Mean age at diagnosis, mean (SD)	72 (10)	66 (11)	66 (9)	62 (12)	< 0.001	
Person‐years followed after diagnosis	2716	123	328	127		
Number of deaths	384	6	33	5		
10‐year survival, % (95% CI)^b^	19.4 (14.4–24.9)	73.3 (47.2–88.0)	30.6 (13.1–50.2)	62.0 (27.1–83.9)	< 0.001	0.001
LBD						
Number of persons	9602	205	622	161		
Women, *n* (%)	5034 (52)	75 (37)	122 (20)	33 (20)	< 0.001	
Mean age at diagnosis, mean (SD)	74 (11)	58 (11)	67 (12)	55 (10)	< 0.001	
Person‐years followed after diagnosis	33,040	1411	2104	913		
Number of deaths	5991	37	283	39		
10‐year survival, % (95% CI)^b^	16.6 (15.5–17.8)	75.8 (66.4–85.9)	23.9 (17.1–31.4)	64.9 (52.7–74.7)	< 0.001	< 0.001

Abbreviations: AD: Alzheimer's disease; CI: confidence interval; FTD: frontotemporal dementia; LBD: Lewy body dementias; SD: standard deviation.

^a^ For age and sex.

^b^ Kaplan‐Meier estimate.

### 3.2 Register Data

#### 3.2.1 Information on Diagnostic Classifications, Crimes, and Mortality

The diagnoses and criminal data were collected between 1998 and 2015. The data collection period for death statistics was extended until the end of 2018. Information between the different registers was linked by the individual identification number assigned to every person registered in Finland.

The information on diagnoses was received from the Finnish Care Register for Health Care maintained by the Finnish Institute for Health and Welfare. The register collects data from all public healthcare providers (hospitals, specialized outpatient services, primary care services, and home nursing service providers) and contains different health and medical data for patients, such as treatment episodes and diagnoses [12]. Classification of diagnoses was based on the 10^th^ revision of the International Statistical Classification of Diseases and Related Health Problems (ICD‐10) [13]. All cases with ICD‐10 diagnosis of AD (F00 and/or G30 codes), FTD (F02.0 and/or G31.0 codes) and LBD, including both Parkinson's disease dementia (F02.3 and/or G20 codes) and dementia with Lewy bodies (F02.8 and/or G31.8 codes), were included. Cases with other types of dementia, such as vascular dementia, dementia of unknown origin or head injuries, were excluded due to their heterogenic nature.

Data on crimes were obtained from the Finnish National Police Register, a nationwide electronic database maintained by the Finnish Police Administration. The Finnish National Police Register (Patja) is part of the police's national information system, which records information about crimes that have occurred, as well as criminal reports, preliminary investigations and all cases in which the police had suspected an individual of a crime [14, 15]. Crime occurrences were monitored during 4 years before and 4 years after diagnosis, until death or until the end of the follow up period. The cumulative incidence and risk of the first post‐diagnosis criminal incidence were assessed over an extended 10‐year follow‐up period to further elucidate the occurrence of criminal behaviour.

As prescribed in greater detail in our previous two studies we found that most types of crimes have been found to be minor crimes, such as traffic and property crimes [5, 9].

Data on 10‐year mortality were collected from Statistics Finland, which covers statistics on deaths of persons permanently domiciled in Finland on the day of death [16].

### 3.3 Statistical Analysis

The descriptive statistics were presented as means with standard deviations (SD) and as counts with percentages (%). Crude or age and sex standardized estimates of crime incidence or incidence rate ratios (IRRs) were calculated using Poisson regression models or random‐effects negative binomial regression models, as appropriate. Poisson regression fits models of the number of counts of an event (crimes). Random‐effects negative binomial regression models used when analysing (“repeated measures”) count data that exhibits overdispersion and when there are individual or group‐specific characteristics that influence the outcome variable. The assumptions of overdispersion in the Poisson model were tested using Lagrange multiplier test. Cumulative rate of crimes in the different groups were estimated using the Kaplan‐Meier product‐limit method method and compared between groups with the Log‐Rank test and Cox regression models. We used also the Fine and Grey competing risks regression model to calculate sub‐hazard ratios (sHR) due to incidence of the crimes (with death as a competing event). Competing‐risks regression models estimate “adjusted” sub‐hazard ratios. The major difference between Competing‐risks model and classical time‐to‐event models is that the risk set for the endpoint of interest includes not only those who have not yet failed because of the primary endpoint but also those who have failed because of a competing cause. Age and sex were used as covariates in these models when appropriate. The statistical significance of the product terms was evaluated using a Wald test. Stata 18.0 (StataCorp LP, College Station, TX, USA) was used for statistical analyses.

## 4 Results

Patients were divided into four subgroups: those without any criminal activity at all (“non‐offenders”); those who committed crimes only before diagnosis (“discontinuers”) or only after diagnosis (“post‐diagnosis offenders”), and those with criminal behaviour both before and after diagnosis (“continuers”) (Table 1).

### 4.1 Criminal Activity in Patients With Neurocognitive Disorders

Most of the patients—75,556 out of 80,540 in the AD group, 899 out of 1059 in the FTD group, and 9602 out of 10,590 in the LBD group—did not show any criminal behaviour.

Most of the offenders committed crimes only before diagnosis (discontinuers), with 4016 in AD, 116 in FTD and 622 in LBD. A small portion—618 AD patients, 22 FTD patients, and 205 LBD patients—started to commit crimes only after diagnosis (post‐diagnosis offenders). A minority of the offenders continued to commit crimes both before and after diagnosis (continuers). There were 350 continuers in the AD group, 22 continuers in the FTD group and 161 continuers in the LBD group.

### 4.2 Sex, Age and Survival

Most patients who engaged in criminal activity were men. This was true for all dementia subtypes and all subgroups with criminal activity (“discontinuers”, “post‐diagnosis offenders” and “continuers”). The percentage of women was significantly higher only in the “non‐offender” group (Table 1).

In all dementia subtypes, patients who committed no crimes at all were significantly older than those with any criminal activity. The subgroup of patients who committed crimes both before and after diagnosis was the youngest across all dementia subtypes (Table 1).

The 10‐year survival, adjusted for age and sex, was significantly lower in the “non‐offenders” and “discontinuers” subgroups compared to patients who committed crimes after diagnosis (“post‐diagnosis offenders” and “continuers”). In “non‐offenders” and “discontinuers”, the lowest rates of 10‐year survival were seen in AD, followed by LBD and FTD. In patients with criminal activity after diagnosis (“post‐diagnosis offenders” and “continuers”), highest survival rates were seen in LBD, followed by FTD and AD (Table 1).

### 4.3 Crime Incidence After Diagnosis of a Neurocognitive Disorder

Patients who committed crimes before diagnosis had a markedly higher incidence of criminal behaviour in the 4 years after diagnosis, compared to those without a prior criminal history. This pattern was consistent across all dementia subtypes. Table 2 shows the incidences of crimes (adjusted for age and sex) in the 4‐year follow‐up period after diagnosis, stratified by the dementia diagnosis (AD, FTD or LBD) and criminal activity before diagnosis. As demonstrated by the incidence rate ratio (IRR), the incidence of crimes after diagnosis was almost sixfold in AD and LBD patients and about fourfold in FTD patients, when comparing patients with criminal activity before diagnosis to those without such criminal history (Table 2).

**TABLE 2:** Age and sex adjusted crime incidences in the 4 years after diagnosis, according by dementia subtypes and criminal activity before diagnosis.

	Criminal activity before diagnosis		
	Yes	No	IRR (95% CI)
	Incidence^a^ (95% CI)	Incidence^a^ (95% CI)	
AD	19.6 (16.0–23.2)	3.4 (3.0–3.9)	5.68 (4.37–7.39)
FTD	47.0 (20.0–73.9)	11.2 (6.1–16.3)	4.18 (2.01–8.71)
LBD	52.9 (40.6–65.2)	9.2 (7.5–10.8)	5.78 (4.26–7.84)

Abbreviations: AD: Alzheimer's disease; CI: confidence interval; FTD: frontotemporal dementia; IRR: incidence rate ratio; LBD: Lewy body dementias.

^a^ Incidence per 1000 person‐years.

### 4.4 Cumulative Incidence of First Post‐Diagnosis Crimes

**FIGURE 1:**
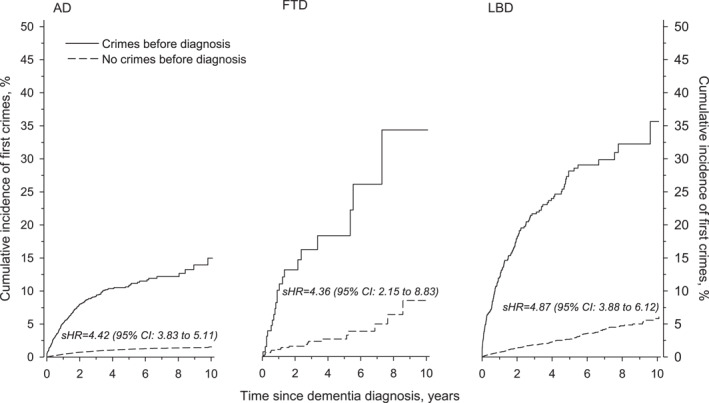
Cumulative incidence of the first post‐diagnosis crime. Adjusted (age and sex) sub‐hazard ratios (sHR) calculated using death as a competing‐risk event. AD: Alzheimer's disease; CI: confidence interval; FTD: frontotemporal dementia; LBD: Lewy body dementias.

The cumulative incidence of the first post‐diagnoses crimes was higher in pre‐diagnosis offenders than in pre‐diagnosis non‐offenders among all dementia subtypes (Figure 1). The steepest rise in the incidence of the first crime occurred in the first two years after diagnosis, but the cumulative incidence continued to increase for the entire 10‐year follow‐up period. We calculated the age‐ and sex‐adjusted subhazard ratio (sHR), which reflects the relative likelihood of the occurrence of the first crime while considering death as a competing risk. The sHRs shown in Figure 1 indicate that the patients who committed crimes before diagnosis were about 4–5 times more likely to commit a first post‐diagnosis crime than patients without a criminal history before diagnosis.

### 4.5 The Risk of First Offence After Diagnosis

We used a competing risk regression model to estimate the Sub‐hazard ratio (sHR) for the risk of the first post‐diagnosis crime, accounting for death as a competing event and adjusting for age and sex. We used the subgroup of patients with no criminal history as the reference group (no crimes at all), with the sHR of 1. We calculated the sHRs separately for patients who had committed crimes in the one, two, three, or 4 years prior to the diagnosis of AD, FTD or LBD. Among all dementia subtypes, the risk of the first post‐diagnosis crime was higher if the patient's pre‐diagnostic criminal activity took place closer in time to the diagnosis. This association was particularly strong when the previous crime occurred within 1 year before diagnosis. For example, when the crime had taken place within 1 year before diagnosis, the risk was nearly seven‐fold in AD (sHR 6.90, 95% CI 5.71–8.35), over four‐fold in FTD (sHR 4.62, 95% CI 1.91–11.15), and over eight‐fold in LBD (sHR 8.42, 95% CI 6.16–11.50) compared to those with no criminal history. When the crime had occurred 2 years prior to diagnosis, the risk was lower but still elevated: sHRs were 3.89 (95% CI 3.05–4.95) in AD, 4.55 (95% CI (1.81–11.42) in FTD, and 4.00 (95% CI 2.75–5.81) in LBD. After that, the association gradually diminished over time and criminal activity that took place three or 4 years before diagnosis was still linked to increased post‐diagnostic risk, but less strongly than more recent crimes.

## 5 Discussion

In this study, most of the diagnosed patients did not exhibit criminal behaviour before or after diagnosis. The majority of those who committed crimes did so only before diagnosis. New‐onset criminal activity after the diagnosis of a neurocognitive disorder was rare. Similarly, only a small segment of the study population had persistent criminal activity both before and after diagnosis.

Even though criminal incidents were rare in absolute numbers, we observed that patients who committed crimes before diagnosis also had a higher post‐diagnosis crime rate. There were differences in the relative risk in post‐diagnosis crimes: in patients who committed crimes before diagnosis, the incidence of criminal behaviour after diagnosis was four times higher in those with FTD and nearly six times higher in those with AD and LBD patients, compared to those with no prior criminal history. The cumulative incidence of the first post‐diagnosis crime was significantly higher in patients who had committed crimes prior to their diagnosis compared to those who had not. The cumulative incidence of the first post diagnosis crime increased throughout the 10‐year follow‐up period, with the steepest rise occurring during the first two years after diagnosis. Criminal activity closer in time to diagnosis, especially the 1 year before the diagnosis, was the most crucial in predicting the likelihood of further criminal activity.

Criminal offenders in our study were predominantly men, which is in line with the general gender difference in crime statistics [17, 18]. The mean age at diagnosis was lower in all the offender groups compared to non‐offenders. Despite the underlying neurocognitive disorder, these younger individuals might still have better functional and physical capacity, overall health, or social connections. This could not only enable them to commit crimes, but also might make their aberrant behaviour more noticeable to others [8]. Neurocognitive disorders differ in their usual age of onset: LBD and FTD typically develop at a younger age, whereas AD has an older median age [19]. These differences were also observed in our study population, with AD patients having a higher mean age at diagnosis than FTD and LBD groups. Early‐onset neurocognitive disorders may also be associated with a different symptom profile compared to late‐onset disorders. This is exemplified by Alzheimer's disease, where younger age of onset has been linked to an increased risk of behavioural and psychological symptoms of dementia [20, 21, 22]. The neuropsychiatric symptoms such as agitation or aggression in part may contribute to an increased likelihood of criminal risk behaviour [23].

Our finding that criminal activity occurred mainly before diagnosis aligns with previous studies showing that criminal behaviour emerges in the earlier stages of neurocognitive diseases [5, 6, 7]. Criminality rates in AD and FTD prior to diagnosis have been shown to be even higher than in the general population of the same age and sex [5], although criminal behaviour is generally rarer among older persons compared to younger population [24, 25, 26]. Novel criminal activity in older age may thus be one of the first symptoms of a neurocognitive disorder [5, 6, 27] and may indicate cognitive and behavioural changes associated with the underlying neurocognitive disorder [27, 28].

The 10‐year survival rates were significantly lower in patients who had no criminal activity after diagnosis (“non‐offenders” and “discontinuers”) compared to those who committed crimes after diagnosis (“post‐diagnosis offenders” and “continuers”). This might be due to a more advanced stage of the disease, which may both prevent criminal activity and shorten the life expectancy [29]. Thus, the progression of the diseases, the increased mortality, but also entering in treatment and care may contribute to the lower amount of crimes after diagnosis [7, 9]. Also, legal consequences such as the revocation of a driver's licence or confiscation of a firearm, may reduce post‐diagnosis criminal behaviour. Although non‐Alzheimer´s dementias may be associated with lower survival, the lower 10‐year survival in our study in the Alzheimer's group probably reflects their higher age at diagnosis [29, 30].

When we focussed only on the group of “continuers”, we found out that criminal activity before diagnosis was a risk factor for its continuation after diagnosis. Crimes committed closer (1 year) to the diagnosis were more predictive of future criminality than those committed further in the past (3–4 years before diagnosis). This finding might reflect the exacerbation of neurocognitive and behavioural symptoms, which become more pronounced and visible and might manifest as an increase in criminal behaviour and then lead to a diagnosis in the following years.

Criminal behaviour during the course of neurocognitive disorders has been previously demonstrated to be recurrent and repetitive to some extent [8]. FTD has been shown to have a higher rate of recurrence of criminal behaviour than AD, possibly related to the frontal lobe damage and difficulties in impulse control typical of FTD [8]. Certain behavioural tendencies in dementia may be persistent. For example, physical aggression is repetitive and continues throughout the progression of the disorder once it has occurred [31, 32]. Thus, the persistence of criminal behaviour throughout the disorder in the group of the continuers may base on persistent and recurrent behavioural traits or patterns.

This study showed differences in the criminal activity of patients with different dementia subtypes. As regards absolute frequences, criminal behaviour at some stages of the disorder was most common in FTD, followed by LBD, and least common in AD. Criminal incidence after diagnosis as well as the cumulative incidence of the first post‐diagnosis crime were higher in FTD and LBD patients compared to individuals with AD. However, in our study, the different groups were kept separate and between‐groups differences were not statistically compared with each other. In the risk relations all diagnostic groups had similar tendencies.

The strengths of our study were the nationwide comprehensive registers with a large number of cases and a long follow‐up period. This enabled a longitudinal analysis of the different patterns of crimes in the course of the neurocognitive disorder. The data encompasses all individuals diagnosed with AD, FTD or LBD in Finland as well as all recorded criminal activity throughout the follow‐up period. Finnish population‐wide registers are considered reliable [33] and accurate [34, 35]. The Finnish Police Register serves as a reliable and impartial source of suspected criminal offences. It is more objective than self‐reported data and more comprehensive than court records, which only cover convictions. Although some crimes might not lead to a conviction, they are still a fairly good indicator of who in the population actually commits crimes. Finnish Care Register for Health Care diagnoses have been recorded using the ICD‐10 system since 1996, therefore the diagnostics has been relying on the international diagnostic criteria, also included in the Finnish current guidelines system [34, 36]. In general, dementia diagnoses recorded in Finnish health care registers are considered highly accurate [35]. Over the study period (1998–2015), diagnostic practices and accuracy have likely improved, particularly in specialist care settings. Diagnostic accuracy is also presumed to be higher in specialist care than in primary health care.

Our study had limitations related to the register‐based design. Underreporting of the crimes may occur in this patient group, as family members or caregivers may not report criminal behaviour to the police [8, 32, 37]. It is also possible that some incidents were not officially recorded by the police due to the presence of obvious cognitive impairment. Individual clinical records were not accessed, so the exact information concerning the diagnostic protocol, stage of the disease, cognitive level or possible neuropsychiatric symptoms were not known. Nor was there a neuropathological confirmation of the diagnosis [12, 16], as differential diagnostics between a primary psychiatric disorder and a neurodegenerative disease may be challenging [38, 39]. Due to the characteristics of this register study, various possible confounding factors, including somatic or psychiatric comorbidities, medication, personality traits, previous lifetime criminal behaviour, or socioeconomic factors, were not controlled for. For individuals exhibiting post‐diagnosis criminal behaviour, it is important to consider the role of treatment and medication, which warrants closer examination in future studies. Thus, multiple potential risk factors of criminal activity in persons with dementia remain unidentified and will require further research.

## 6 Conclusion

According to our results, criminal behaviour in persons with dementia is quite rare, even though dementias affect cognition, behaviour, reasoning, and judgement abilities and often cause neuropsychiatric symptoms. Criminal activity appears to be more prevalent in the period leading up to a dementia diagnosis rather than afterwards. If new‐onset criminal behaviour occurs in an older person, the possibility of an undiagnosed neurocognitive disorder should be kept in mind.

A small minority of our study population continued their criminal activity after the diagnosis of a neurocognitive disorder, and this subgroup could be identified by criminal acts committed close in time before diagnosis. The year leading up to the diagnosis was the most critical in terms of the increase in the relative risk of further criminal activity.

For individuals who had committed crimes before their diagnosis, the risk of committing crimes increased after diagnosis, particularly within the first 2 years. This higher risk and the cumulative incidence of crimes remained elevated even beyond this period, and this should be taken into account in the treatment of this population.

## Author Contributions

All authors took part in study conception, design, final approval, and agreement to be accountable, M.G., T.T., H.Ka., A.P., H.Ko. and N.L. also in draughting, H.Ka. and H.Ko. in acquisition, and H.Ka. in analysis. Critical revision was performed by R.V., H.E. and J.S.

## Ethics Statement

The study protocol was approved by the Coordinating Ethics Committee of Helsinki University Hospital (no. 186/13/03/00/16).

## Consent

The study was conducted as a register study and did not involve contacting the participants. Thus, no informed consent was required.

## Conflicts of Interest

The authors declare no conflicts of interest.

## Permission to Reproduce Material From Other Sources

The authors have nothing to report.

## Data Availability

The data related to this paper are obtained from access to national health registries via Statistics Finland, the Finnish Institute for Health and Welfare and the Police of Finland, and the authors cannot be authorized to transfer data to other researchers.
